# Supplementary material for: Phase-Controllable Chemical Vapor Deposition Synthesis of Atomically Thin MoTe2
Source: Nanomaterials (Basel). 2022 Nov 23;12(23):4133. doi: 10.3390/nano12234133 (PMC9737202; doi:10.3390/nano12234133)
Supplement: Supplementary file 1 [file nanomaterials-12-04133-s001.zip › nanomaterials-2038329-supplementary.pdf]

nanomaterials

Supplementary Materials for:

Phase-Controllable Chemical Vapor Deposition Synthesis of Atomically Thin $MoTe_2$

Tao Xu [1], Aolin Li [2], Shanshan Wang [3], Yinlong Tan [1] and Xiang'ai Cheng [1,*]

[1] College of Advanced Interdisciplinary Studies, National University of Defense Technology, Changsha 410073, China
[2] Powder Metallurgy Research Institute, Central South University, Changsha 410073, China
[3] Science and Technology on Advanced Ceramic Fibers and Composites Laboratory, College of Aerospace Science and Engineering, National University of Defense Technology, Changsha 410073, China
* Correspondence: xiang_ai_cheng@126.com

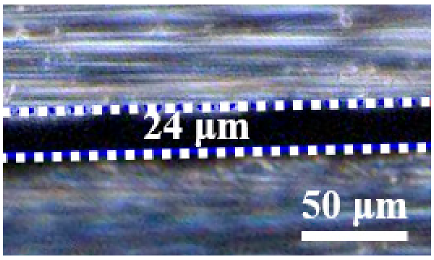

Figure S1. Optical images displaying the gap size of the stacked SiO_2/Si substrates in a face-to-face way.

The gap size of confined space by the face-to-face SiO_2/Si substrates was measured by the ultra-depth three-dimensional microscope. Notably, the lens of the microscope must be rotated 90° horizontally to observe the size of the gap.

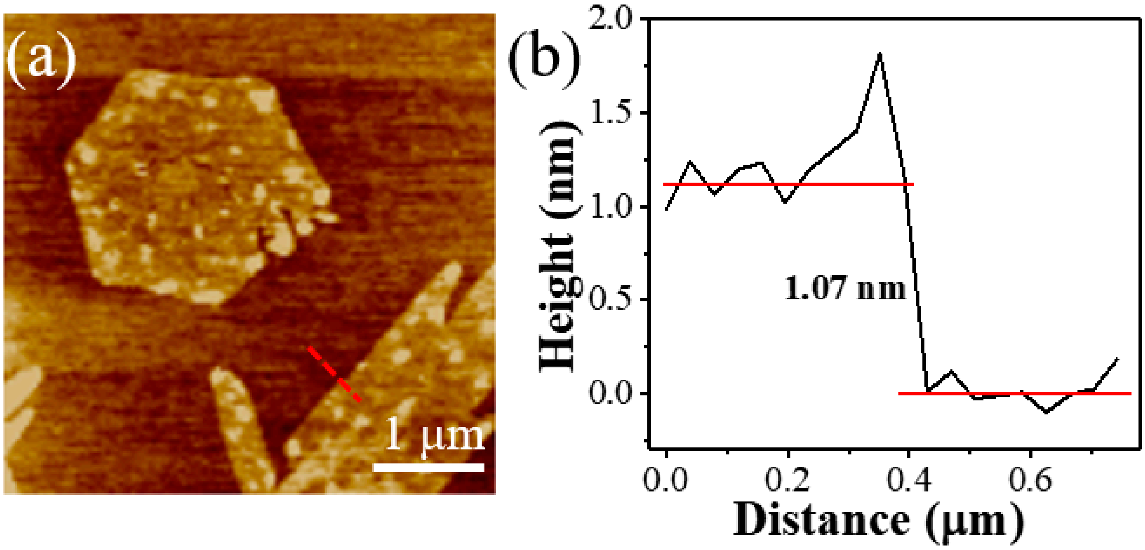

Figure S2. AFM image of monolayer $MoTe_2$ grains and its corresponding height profile.

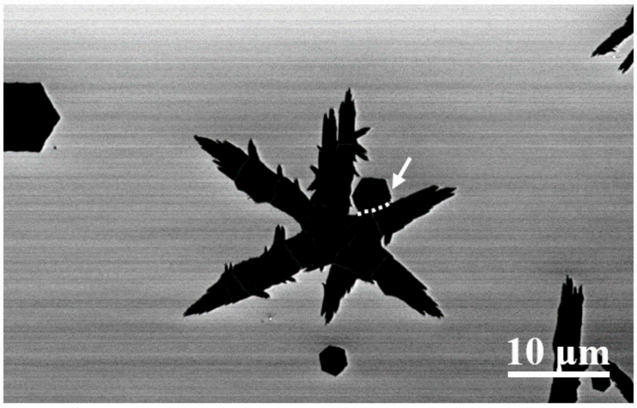

Figure S3. SEM image of as-synthesized 2H and 1T′ $MoTe_2$ homojunction.

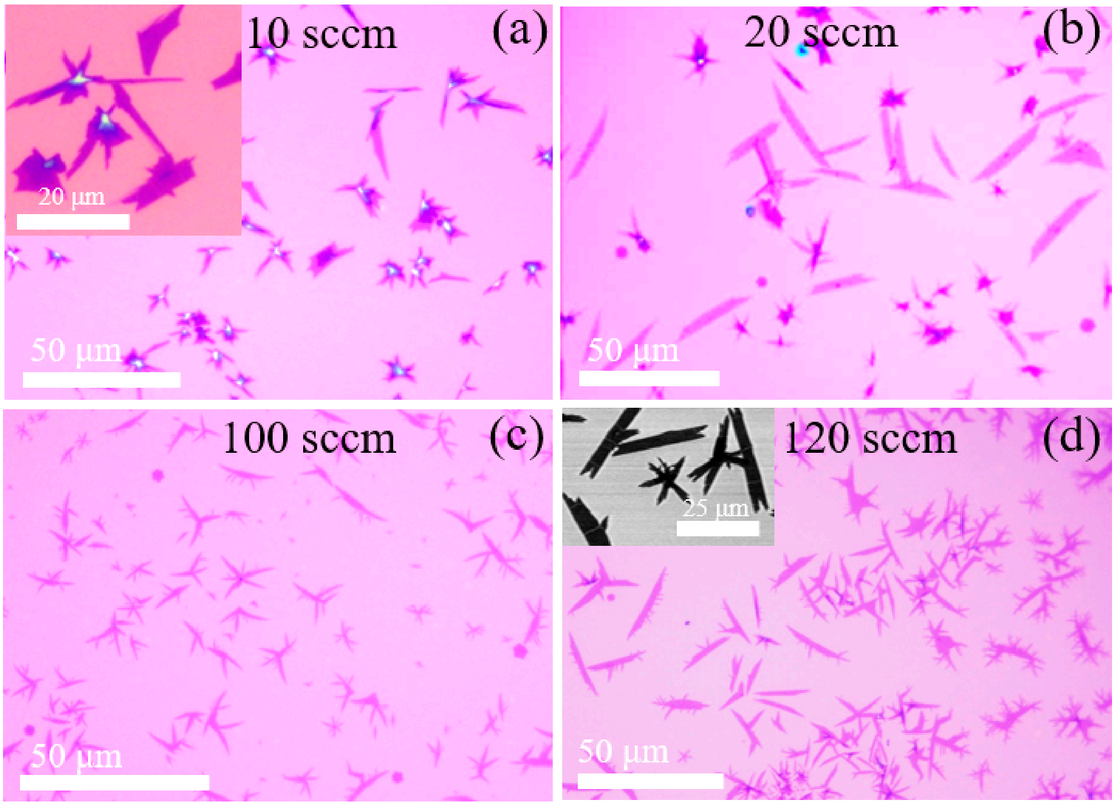

Figure S4. (a-d) Optical images of as-synthesized few-layer $MoTe_2$ under different gas flow rates of 10 sccm, 20 sccm, 100 sccm, and 120 sccm, respectively.

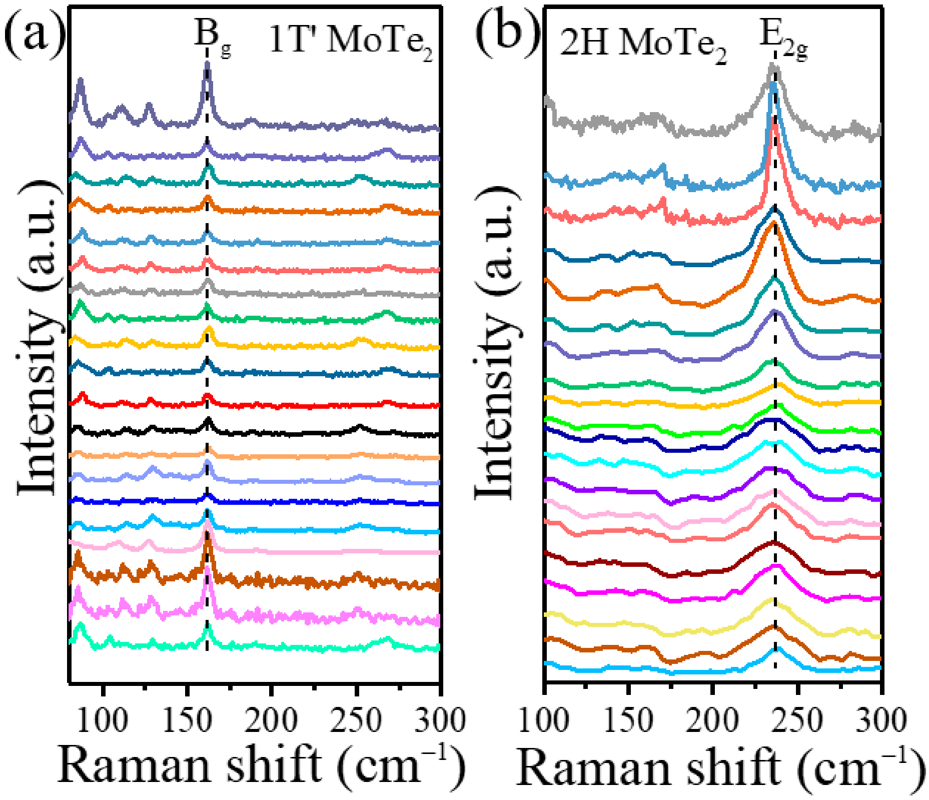

Figure S5. (a-b) Raman spectra of 20 hexagonal grains and 20 leaf-like grains, respectively.

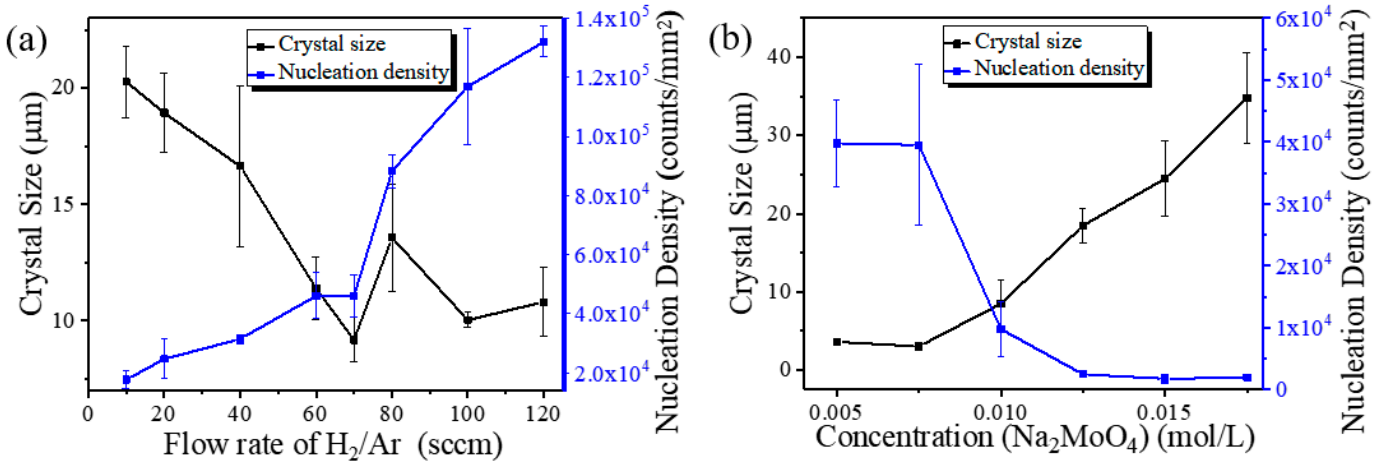

Figure S6. (a) Plot of the variation trend of crystal size and nucleation density as a function of the gas flow rate. (b) Plot of the trend of crystal size and nucleation density as a function of the concentration of Na_2MoO_4 solution.

It is obvious that the rising concentration of the Na_2MoO_4 solution could reduce the nucleation density but boosts the average crystal size, as shown in Figure S6b, which is indeed contrary to the trend of the nucleation density and crystal size as the function of Te concentration controlled by the gas flow rate (Figure S5a).

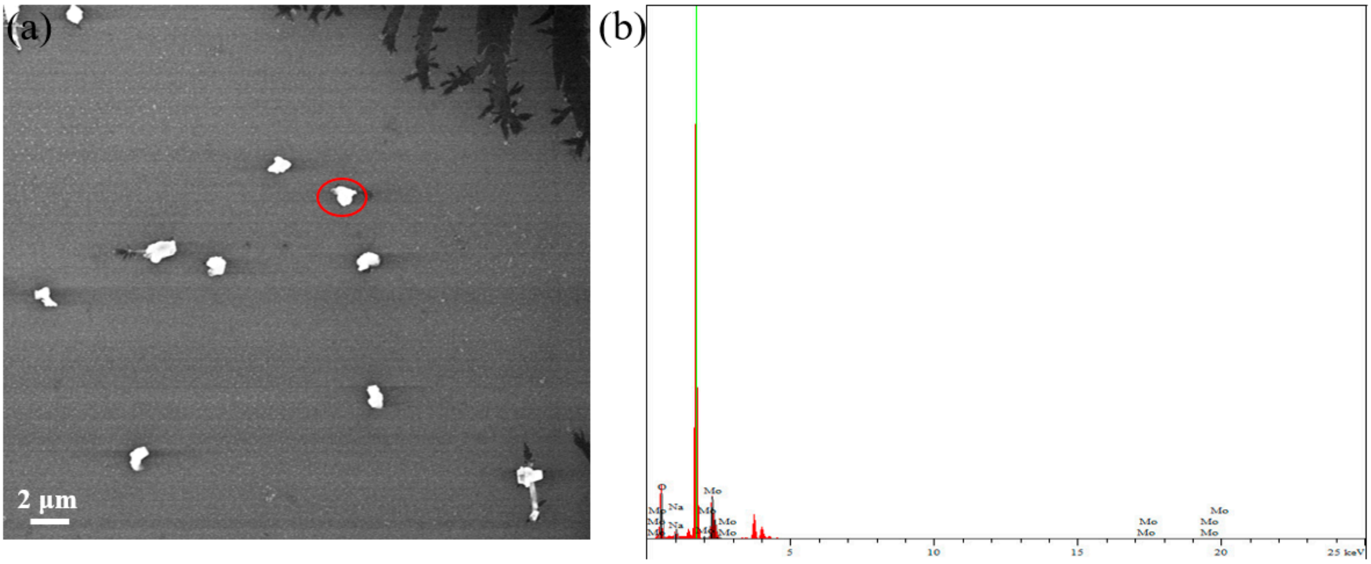

Figure S7. (a-b) SEM image and Energy Dispersive Spectroscopy of the nanoparticles related to Na_2MoO_4, respectively.

In our density functional theory (DFT) calculations, we have used supercells to simulate the monolayer MoTe2 with Te vacancies, as shown in Figure S8. These supercells contain one Te vacancy and 4, 8, 12, 16, 20, 24 unit cells, respectively. Therefore, the Te vacancy concentration is 12.5%, 6.3, 4.2%, 3.1%, 2.5% and 2.1%, respectively.

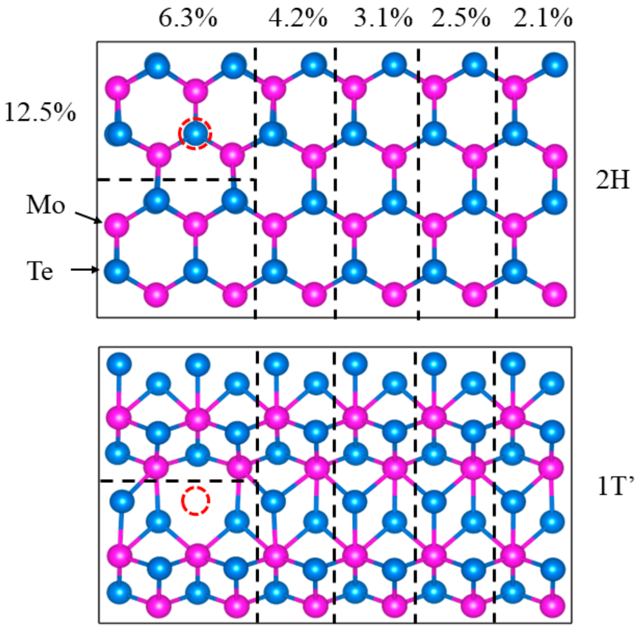

Figure S8. Atomic structures used in the DFT calculations.
